# Beyond Molecular Classification in Metastatic Triple-Negative Breast Cancer: Toward Subtype-Guided Precision Oncology

**DOI:** 10.3390/ijms27115040

**Published:** 2026-06-02

**Authors:** Leonel Pekarek, Cielo García-Montero, Carlos Casanova-Martin, Miguel A. Ortega, Óscar Fraile-Martínez

**Affiliations:** 1 Department of Medicine and Medical Specialities, Faculty of Medicine and Health Sciences, University of Alcala, 28801 Alcala de Henares, Spain; leonel.pekarek@gmail.com (L.P.); cielo.gmontero@gmail.com (C.G.-M.); ccasanovam91@gmail.com (C.C.-M.); oscarfra.7@hotmail.com (Ó.F.-M.); 2 Ramón y Cajal Institute of Sanitary Research (IRYCIS), 28034 Madrid, Spain

**Keywords:** triple-negative breast cancer, metastatic breast cancer, molecular subtypes, biomarkers, precision oncology, antibody–drug conjugates

## Abstract

Metastatic triple-negative breast cancer (mTNBC) remains one of the most challenging therapeutic settings in oncology. Although it has traditionally been defined by the absence of hormone receptor expression—estrogen receptor (ER) and progesterone receptor (PR)—and HER2 amplification or overexpression, this simplified definition fails to capture the biological complexity that drives its marked clinical heterogeneity, therapeutic resistance, and prognostic variability. Over the past decade, multiple studies have challenged the notion of TNBC as a single disease entity, identifying distinct molecular subtypes, including Basal-like 1 (BL1), Basal-like 2 (BL2), Mesenchymal (M), Mesenchymal Stem-like (MSL), Immunomodulatory (IM), and Luminal Androgen Receptor (LAR), each characterized by specific biological programs and therapeutic vulnerabilities. In parallel, clinically oriented systems such as the Fudan classification have enabled the prospective evaluation of subtype-guided therapeutic strategies in metastatic disease, as illustrated by the FUTURE and FUTURE-SUPER trials. In this review, we examine the molecular classification and clinical behavior of mTNBC subtypes, integrating genomic, transcriptomic, epigenetic, immunologic, stromal, and biomechanical dimensions of tumor heterogeneity. We also discuss emerging tools, including single-cell RNA sequencing, spatial transcriptomics, circulating tumor DNA analysis, long non-coding RNA profiling, and surrogate immunohistochemistry-based classifiers, as well as their potential role in refining patient stratification. From a therapeutic perspective, we review subtype-guided strategies involving chemotherapy, platinum agents, PARP inhibitors, immunotherapy, antiandrogen therapy, PI3K/AKT/mTOR pathway inhibition, antiangiogenic approaches, and antibody–drug conjugates. Redefining mTNBC through biologically driven stratification represents a rational strategy to optimize treatment selection, support clinical trial design, and accelerate the development of precision oncology approaches. However, clinical implementation requires greater methodological standardization, validated predictive biomarkers, accessible diagnostic platforms, and dynamic monitoring strategies capable of capturing subtype evolution under therapeutic pressure. TNBC should therefore not be regarded as a single disease, but as a spectrum of biologically distinct and clinically evolving entities whose integrated characterization may be essential to improving outcomes in this historically poor-prognosis population.

## 1. Introduction

### 1.1. Epidemiology and Prognosis of mTNBC in the Current Landscape

Metastatic triple-negative breast cancer (mTNBC) represents one of the most challenging entities in contemporary oncologic practice. This aggressive subtype, defined by the absence of estrogen receptor (ER) and progesterone receptor (PR) expression, as well as the lack of HER2 overexpression or amplification, accounts for approximately 10–15% of all breast cancers and is associated with a particularly poor prognosis in the metastatic setting, along with limited response rates in both neoadjuvant and adjuvant contexts [1,2,3].

The median overall survival (OS) of patients with mTNBC ranges between 8 and 14 months across different series, compared with approximately 5 years observed in hormone receptor–positive or HER2-positive subtype [2,4,5]. Recent data from the National Cancer Database report a median OS of 13.6 months in a cohort of 2273 women with mTNBC [4]. Real-world evidence from the United States corroborates these findings, showing a median OS of 14.0 months from diagnosis, with a progression-free survival (PFS) of only 4.5 months in the first-line setting and 4.1 months in the second-line setting [6]. These data underscore the marked aggressiveness of this disease. Notably, unlike HER2-positive and hormone receptor–positive breast cancers—where the incorporation of novel therapies has substantially improved outcomes—survival gains in TNBC have been considerably more modest.

Five-year survival rates further highlight the severity of this entity: while survival reaches 91.3% in localized disease, it declines to 65.8% in regional disease and drops to just 12.0% in metastatic disease [1,6]. This dramatic reduction underscores the urgent need for more effective therapeutic strategies and for refined subclassification systems to better guide treatment decisions, particularly considering that the median age at diagnosis is around 50 years.

Chemotherapy remains the cornerstone of treatment in mTNBC, although its efficacy is modest, with objective response rates (ORR) of approximately 23% in the first-line setting and 11% in subsequent lines [7,8]. Moreover, responses are typically short-lived, with median durations of 4.4–6.6 months in the first-line setting and 4.2–5.9 months in second-line or later lines [7,8]. A pooled analysis of phase III trials reported a median OS of 17.5 months with first-line chemotherapy, decreasing to approximately 12 months in later lines [7].

Importantly, approximately 50% of patients with mTNBC do not receive second-line treatment, primarily due to death from rapidly progressive visceral disease (approximately 30% of cases) or clinical deterioration precluding further therapy [3,7]. This clinical reality emphasizes the critical importance of optimizing therapeutic decision-making from the outset.

### 1.2. Limitations of the Current Receptor-Based Classification (ER-/PR-/HER2-)

The current definition of TNBC, based on the absence of ER, PR, and HER2 expression, represents a classification by exclusion that, although clinically practical, fails to capture the true biological complexity of these tumors [9]. This designation provides a convenient framework to group a heterogeneous set of neoplasms, yet it does not adequately reflect their underlying biology nor reliably predict therapeutic response. Essentially, it clusters tumors under a single label based solely on the absence of classical biomarkers, without accounting for tumor diversity or differences in prognosis and treatment sensitivity among patients [9].

A key limitation is that the terms “triple-negative” and “basal-like” are not synonymous, with a discordance of approximately 25–30% between molecular classification and immunohistochemistry based on the three standard markers [10,11]. Approximately 80% of TNBCs are basal-like, and most basal-like breast cancers (around 80%) are also TNBC; however, clinical, microarray, and immunohistochemical data demonstrate that this overlap is incomplete [10]. The term “basal-like” is defined by gene expression profiles that recapitulate features of basal or myoepithelial cells of the mammary gland. It is typically associated with high-grade tumors, rapid proliferation, and aggressive clinical behavior. In clinical practice, most basal-like tumors are triple-negative and express basal markers such as CK5/6 and EGFR. This subtype is more common in younger women, is associated with a high rate of early relapse, and is frequently linked to defects in DNA repair pathways, particularly BRCA1 mutations, which influence sensitivity to chemotherapy and targeted therapies in specific contexts.

Beyond basal-like tumors, TNBC encompasses additional molecular subtypes, including claudin-low tumors—enriched in stem cell–like features and epithelial–mesenchymal transition (EMT) characteristics—the interferon-rich subgroup, which is associated with a more favorable prognosis, and the normal breast-like subgroup [10].

Current thresholds for hormone receptor positivity also influence classification. Updated guidelines have lowered the cutoff for ER and PR positivity from 10% to 1%, increasing the number of patients eligible for endocrine therapy but potentially reducing the number classified as TNBC [9]. Gene expression analyses have shown that 76% of tumors with low hormone receptor expression (1–9%) are ESR1-negative at the mRNA level, and 48% are classified as basal-like [9]. This has led to the proposal of a new category termed “triple-negative-like breast cancer” (TN-like BC), which may require distinct therapeutic strategies compared to conventional TNBC [12].

From a genomic perspective, TNBC is highly heterogeneous. Multiple recurrent genomic amplifications have been identified, although the individual prevalence of each alteration is low [10]. This genomic diversity contributes to the inconsistent responses and variable outcomes observed with current therapeutic approaches, which continue to be associated with limited and heterogeneous response rates [13]. Consequently, a classification based solely on receptor status fails to identify specific molecular vulnerabilities or to guide optimal selection of targeted therapies in a disease defined more by the absence of biomarkers than by a true understanding of tumor biology.

### 1.3. Molecular Subclassification in the Metastatic Setting

**Figure 1:**
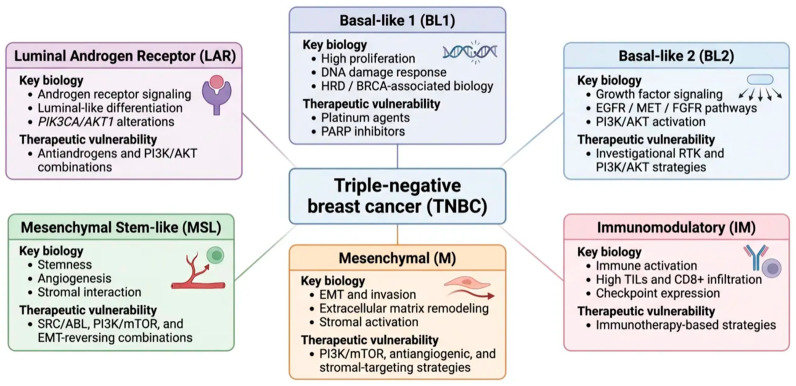
Molecular heterogeneity and major subtypes of triple-negative breast cancer. The figure summarizes the principal biological, molecular, and therapeutic features of the main TNBC subtypes according to the classical Lehmann classification. BL1 is characterized by high proliferation, genomic instability, activation of DNA damage response pathways, and frequent homologous recombination deficiency, supporting sensitivity to platinum agents and PARP inhibitors in selected patients. BL2 shows enrichment in growth factor signaling pathways, including EGFR, MET, FGFR, and PI3K/AKT-related programs. The Mesenchymal subtype is associated with epithelial–mesenchymal transition, extracellular matrix remodeling, stromal activation, mechanotransduction, and relative chemoresistance. The Mesenchymal Stem-like subtype displays stemness-related signatures, angiogenesis, EGFR signaling, and stromal influence. The Immunomodulatory subtype is enriched in immune activation, tumor-infiltrating lymphocytes, interferon signaling, and immune checkpoint expression, supporting sensitivity to immunotherapy-based strategies. The Luminal Androgen Receptor subtype is defined by androgen receptor signaling, luminal-like differentiation, frequent PIK3CA/AKT1 alterations, and lower sensitivity to conventional chemotherapy. The figure highlights that TNBC should be regarded as a spectrum of biologically distinct entities rather than a single disease. Abbreviations: AR, androgen receptor; BL1, Basal-like 1; BL2, Basal-like 2; EGFR, epidermal growth factor receptor; EMT, epithelial–mesenchymal transition; FGFR, fibroblast growth factor receptor; HRD, homologous recombination deficiency; IM, Immunomodulatory; LAR, Luminal Androgen Receptor; M, Mesenchymal; MET, mesenchymal–epithelial transition factor; MSL, Mesenchymal Stem-like; PARP, poly(ADP-ribose) polymerase; TILs, tumor-infiltrating lymphocytes; TNBC, triple-negative breast cancer.

One of the principal challenges in the management of TNBC lies in its molecular heterogeneity, which largely accounts for the inconsistent responses and variable outcomes observed with existing therapeutic approaches, often characterized by poor response rates and an unfavorable prognosis [13]. Several molecular subclassification systems have been proposed, including those developed by Lehmann and colleagues, based on gene expression profiling to distinguish biologically distinct subtypes [13]. These models have enhanced our understanding of TNBC biology and have driven clinical research, although their applicability in routine clinical practice has historically been limited. The molecular complexity of TNBC can be organized into recurrent biological subtypes with distinct signaling programs and potential therapeutic vulnerabilities (Figure 1).

In recent years, molecular profiling has enabled the identification of TNBC subtypes with distinct biological, prognostic, and therapeutic characteristics. The seminal work by Brian D. Lehmann initially classified TNBC into six subtypes, later refined to four by subsequent studies, as the IM and MSL subtypes are now considered to primarily reflect tumor microenvironmental features rather than intrinsic tumor cell phenotypes [14,15]. From a classical molecular standpoint, the six principal subtypes include: Basal-like 1 (BL1) and Basal-like 2 (BL2), characterized by high expression of genes involved in cell cycle regulation and DNA damage response; Immunomodulatory (IM), marked by high lymphocytic infiltration and immune-related gene expression; Mesenchymal (M) and Mesenchymal Stem-like (MSL), associated with activation of multiple growth and proliferation pathways, with MSL showing enhanced expression of stemness-related genes and EGFR signaling; and Luminal Androgen Receptor (LAR), characterized by androgen receptor expression and activation of hormonally regulated pathways [9,14,16].

Each subtype exhibits a distinct mutational landscape with specific therapeutic implications. The LAR subtype is associated with mutations in PIK3CA (55%), AKT1 (13%), and CDH1 (13%), suggesting potential sensitivity to PI3K pathway inhibitors [14,15]. The IM subtype shows high expression of immune checkpoint genes (PD-1, PD-L1, CTLA4), supporting a potential role for immunotherapy [14,15]. The BL1 subtype is characterized by high genomic instability, with frequent TP53 mutations (92%) and deletions in DNA repair genes (BRCA2, PTEN, RB1), indicating sensitivity to DNA-damaging agents [14]. The mesenchymal subtype (MES) shows enrichment in RTK-RAS signaling and may be sensitive to tyrosine kinase inhibitors [16,17].

The clinical relevance of molecular subtyping has been prospectively evaluated in the FUTURE-SUPER trial, a multicohort, randomized phase II study conducted in the first-line setting of mTNBC. In this trial, patients were stratified according to a molecular framework integrating transcriptomic subtypes (LAR, IM, BLIS, and MES) with actionable genomic alterations, and were treated with subtype-specific targeted combinations in addition to nab-paclitaxel. This subtype-informed approach significantly improved progression-free survival (PFS) compared with chemotherapy alone (11.3 vs. 5.8 months; hazard ratio [HR] 0.44; *p* < 0.0001).

Notably, the magnitude of benefit varied across biologically defined cohorts. The greatest improvement was observed in the immunomodulatory subtype (15.1 vs. 6.5 months; HR 0.46), followed by the BLIS/MES-PI3K/AKT wild-type cohort (9.1 vs. 3.9 months; HR 0.35). In the LAR-PI3K/AKT-mutant subgroup, PFS was also prolonged (13.9 vs. 6.1 months; HR 0.48), although this did not reach statistical significance, likely due to limited sample size. In addition to PFS benefit, subtype-based therapy was associated with a higher objective response rate (80.0% vs. 44.8%), reinforcing the biological rationale of this strategy [13].

These findings represent a major advance in the management of TNBC and help explain part of the prognostic variability observed among patients. Unlike previous studies focused on single-gene alterations—where only a small fraction of screened patients were ultimately eligible—this approach classifies the entire TNBC population into defined subtypes and explores tailored therapeutic strategies for each. The results reinforce the potential of molecular subclassification to guide treatment selection in mTNBC and provide a clear direction for future clinical development [13]. Taken together, molecular subclassification of mTNBC represents a true paradigm shift, enabling a transition from a uniform, chemotherapy-based approach to a precision oncology framework aimed at identifying subtype-specific vulnerabilities. In this context, this strategy has the potential to optimize treatment selection, improve clinical outcomes, and ultimately modify the prognosis of patients with this historically challenging disease.

### 1.4. Literature Search Strategy

This narrative review was developed through a structured literature search aimed at identifying relevant studies on the molecular classification, biological heterogeneity, clinical behavior, and therapeutic implications of metastatic triple-negative breast cancer (mTNBC). The literature search was performed using PubMed/MEDLINE, Scopus, Web of Science, and ClinicalTrials.gov. The search primarily focused on articles published between January 2010 and March 2026, while selected earlier landmark studies were also considered when they were essential to define the historical and biological foundations of TNBC classification. The search strategy included combinations of the following terms: “triple-negative breast cancer”, “metastatic triple-negative breast cancer”, “molecular subtypes”, “Lehmann classification”, “Fudan classification”, “Burstein classification”, “basal-like breast cancer”, “luminal androgen receptor”, “immunomodulatory subtype”, “mesenchymal subtype”, “BLIS”, “MES”, “FUTURE trial”, “FUTURE-SUPER”, “precision oncology”, “immunotherapy”, “PARP inhibitors”, “PI3K/AKT/mTOR”, “antibody-drug conjugates”, “sacituzumab govitecan”, “trastuzumab deruxtecan”, “single-cell RNA sequencing”, “spatial transcriptomics”, “long non-coding RNAs”, and “chemoresistance”.

Priority was given to peer-reviewed original articles, clinical trials, translational studies, meta-analyses, systematic reviews, and high-quality narrative reviews published in English. Particular emphasis was placed on studies reporting molecular subtype-specific biology, therapeutic vulnerabilities, clinical outcomes, and biomarker-driven treatment strategies in TNBC or mTNBC. Clinical trial records were reviewed when relevant to ongoing subtype-directed therapeutic strategies. Articles were excluded when they were not focused on TNBC, lacked direct relevance to molecular classification or therapeutic implications, were not available in English, or provided only anecdotal data without clear methodological description. Because this is a narrative review rather than a systematic review, no formal quantitative synthesis or meta-analysis was performed.

## 2. Molecular Heterogeneity of Metastatic TNBC

### 2.1. Genomic and Transcriptomic Diversity in TNBC

Triple-negative breast cancer is characterized by striking genomic and transcriptomic heterogeneity, distinguishing it from other breast cancer subtypes. This molecular diversity is evident across multiple levels, ranging from copy number alterations to differential gene expression patterns, and represents a major barrier to the development of truly effective targeted therapies [17,18].

At the genomic level, TNBC exhibits a relatively moderate mutational burden compared with other solid tumors. The total number of somatic non-synonymous mutations assessed by whole-exome sequencing is higher in TNBC (median of 49 mutations) than in luminal breast cancer (median of 27 mutations), although it remains lower than in malignancies such as melanoma, non-small cell lung cancer, or microsatellite instability–high colorectal cancer [9,18]. The most prevalent mutations include TP53 (50–92%) and PIK3CA (10–55%), with frequencies that vary significantly across molecular subtypes. Alterations in the PI3K/AKT/mTOR pathway are relatively frequent, particularly in LAR and mesenchymal-like tumors; however, clinical benefit from pathway inhibition in TNBC has been inconsistent and remains an area of active investigation [16,19].

Integrated multi-omic analyses have demonstrated that each TNBC molecular subtype harbors a distinct mutational landscape with specific therapeutic implications. The Basal-like 1 (BL1) subtype is characterized by the highest degree of genomic instability, with a high prevalence of TP53 mutations (92%) and deletions in genes involved in DNA repair pathways (BRCA2, MDM2, PTEN, RB1) [16,18]. In contrast, the Luminal Androgen Receptor (LAR) subtype exhibits the highest mutational burden, with significant enrichment of mutations in PIK3CA (55%), AKT1 (13%), and CDH1 (13%) [16,17,18,19]. The Immunomodulatory (IM) subtype shows elevated expression of immune checkpoint genes such as PD-1, PD-L1, and CTLA4, whereas the Mesenchymal (M) and Mesenchymal Stem-like (MSL) subtypes are associated with higher angiogenesis-related gene signatures [16,17,18,19].

The transcriptomic diversity of TNBC has been characterized through multiple classification systems. As previously discussed, the Lehmann classification initially identified six subtypes (BL1, BL2, IM, M, MSL, and LAR), later refined into four principal subtypes. Another relevant framework is the Burstein classification, which proposed four alternative subtypes: Luminal/Androgen Receptor (LAR), Mesenchymal (MES), Basal-like/Immune-Suppressed (BLIS), and Basal-like/Immune-Activated (BLIA) [9,15,20]. Each system captures complementary aspects of TNBC biology, including immune infiltration, proliferative capacity, androgen receptor signaling, and homologous recombination deficiency [20].

Recent studies have further expanded the molecular characterization of TNBC through DNA methylation profiling, identifying epigenetic subtypes corresponding to basal and non-basal groups. These subtypes show distinct transcriptional programs that correlate with DNA methylation patterns in distal regulatory elements and with epigenetic regulation of genes involved in steroid response and developmental transcription factors [8]. The integration of proteomic and phosphoproteomic data has enabled the identification of signaling pathways with potential as subtype-specific therapeutic targets, while single-cell analysis of the tumor microenvironment is emerging as a promising tool to further elucidate TNBC heterogeneity and better explain its biological behavior and clinical aggressiveness [21,22].

In addition to genomic, transcriptomic, epigenetic, and proteomic layers, long non-coding RNAs (lncRNAs) are emerging as relevant regulators of TNBC heterogeneity. lncRNAs can modulate gene expression through chromatin remodeling, transcriptional regulation, RNA splicing, competing endogenous RNA networks, and post-transcriptional control of oncogenic signaling pathways. In TNBC, several lncRNA-associated programs have been implicated in epithelial–mesenchymal transition, cancer stemness, immune evasion, DNA damage repair, metabolic adaptation, and metastatic dissemination. These mechanisms may contribute to the acquisition of subtype-specific phenotypes and to the dynamic transition between basal-like, mesenchymal, and immune-modulated states [23].

lncRNAs may also influence chemotherapy resistance profiles. By regulating apoptosis, drug efflux, DNA repair capacity, autophagy, and EMT-associated plasticity, lncRNA networks can alter sensitivity to anthracyclines, taxanes, platinum compounds, and other cytotoxic agents. Although most evidence remains preclinical or translational, these findings suggest that lncRNAs may function as biomarkers of treatment resistance and as potential therapeutic targets. Their integration into multi-omic TNBC classification could therefore refine current transcriptomic subtype models and help identify biologically defined groups with distinct patterns of therapeutic vulnerability [23].

### 2.2. Intratumoral Heterogeneity and Chemoresistance

Intratumoral heterogeneity in TNBC plays a critical role in therapeutic resistance and represents one of the most complex barriers to effective treatment, contributing to the low response rates observed with currently available therapies. This heterogeneity is evident at both genetic and non-genetic levels and includes transcriptomic and epigenetic alterations that promote immune evasion and drug resistance [24,25].

Single-cell sequencing studies have shown that the main transcriptional subtypes—luminal, basal, and mesenchymal—tend to be relatively homogeneous within individual tumor samples, although substantial interpatient variability exists [23,24,25,26]. The transcription factor PRRX1 has been identified as a key driver of mesenchymal tumors and is sufficient to induce mesenchymal features in basal-type TNBC cells, but not in luminal cells, through reprogramming of the super-enhancer landscape [26].

Recent advances in single-cell RNA sequencing (scRNA-seq) and spatial transcriptomics have further refined our understanding of intratumoral heterogeneity in TNBC. These technologies have shown that TNBC is not only composed of genetically distinct clones, but also of transcriptionally diverse cellular states that may coexist within the same lesion. Malignant cells can display basal-like, mesenchymal, proliferative, interferon-related, or stem-like programs, and these states may shift dynamically under therapeutic pressure. This plasticity provides a mechanistic explanation for why a single bulk transcriptomic subtype may not fully capture the biological complexity of an individual tumor.

Spatial transcriptomic approaches add an additional layer of information by preserving the anatomical organization of tumor cells, stromal elements, immune infiltrates, and vascular structures. These studies suggest that treatment response is influenced not only by the intrinsic phenotype of malignant cells, but also by their spatial proximity to fibroblasts, macrophages, cytotoxic T cells, exhausted lymphocytes, and extracellular matrix-rich niches. In this context, mesenchymal or immune-suppressed regions may coexist with immune-inflamed areas within the same tumor, creating spatially restricted therapeutic vulnerabilities and resistance reservoirs. Incorporating single-cell and spatial data into TNBC classification may therefore improve the ability to predict subtype evolution, immune escape, and resistance to systemic therapy [27].

In this context, clonal evolution under therapeutic pressure is a well-documented phenomenon in metastatic TNBC. Multiple studies and reviews have demonstrated significant heterogeneity in subclonal architecture from primary tumors to metastatic disease, as well as across different metastatic sites, indicating that no single lesion fully captures the complexity of the disease. This supports the existence of highly aggressive clonal lineages capable of driving visceral crisis and contributing to the high mortality associated with this entity [28].

Circulating tumor DNA (ctDNA) analysis has emerged as a valuable tool for real-time monitoring of clonal evolution. A recent multicenter study introduced the concept of tumor evolution rate (TER) as a novel indicator of clonal dynamics [29]. Patients with branched evolutionary patterns exhibited better therapeutic outcomes than those with linear evolution, and individuals classified within the low-TER group demonstrated improved progression-free survival (HR 0.62; 95% CI: 0.40–0.96; *p* = 0.033) and overall survival (HR 0.45; 95% CI: 0.24–0.85; *p* = 0.013) [29].

In parallel, the hypothesis proposed by Ibragimova et al. suggests that chemotherapy may paradoxically accelerate clonal evolution, promoting the emergence of new malignant and resistant clones responsible for metastatic progression [30]. Alternatively, treatment-induced selective pressure may facilitate the expansion of pre-existing minor clones that become dominant following the elimination of chemosensitive populations. According to this hypothesis, chemotherapy-driven clonal selection could contribute to metastatic progression and relapse in a subset of cases; however, this interpretation remains controversial and should be considered exploratory [30].

Furthermore, preliminary analyses of mutational signatures in paired TNBC samples treated with neoadjuvant chemotherapy have identified differences between tumors achieving pathological complete response (pCR) and those with residual disease [31]. A predominance of C>A transversions was observed in tumors achieving pCR, whereas C>T transitions were more frequently identified in primary tumors without pCR and in residual disease samples, suggesting the involvement of distinct mutational processes. Differences in the mutational landscape were also reported, with enrichment of alterations in genes such as BRCA2, TP53, and EP300 in tumors achieving pCR, while mutations in BRCA2, KMT2A, and ROS1 were more commonly detected in non-pCR tumors [31]. In addition, tumor mutational burden appeared higher in primary tumors from patients who did not achieve pCR. However, these findings are derived from a limited exploratory cohort and should be interpreted with caution, requiring validation in larger prospective studies. These observations may reflect differences in tumorevolutionary trajectories under treatment pressure, including clonal selection and adaptive resistance mechanisms [31].

### 2.3. Limitations of the “Triple-Negative” Label for Therapeutic Guidance

The current classification of TNBC, based on the absence of ER, PR, and HER2 expression, represents a definition by exclusion that, although useful for initial clinical decision-making, fails to reflect the biological complexity of these tumors and does not enable optimal therapeutic selection [32].

A major limitation lies in the discordance between immunohistochemical and molecular classifications. The terms “triple-negative” and “basal-like” are not interchangeable: approximately 21% of TNBCs are not basal-like, and 31% of basal-like tumors are not triple-negative [3,22,32]. This discordance has direct clinical implications, as different molecular subtypes exhibit distinct responses to both chemotherapy and targeted therapies. The “triple-negative” designation does not identify specific molecular vulnerabilities nor reliably predict therapeutic response. While these patients do not benefit from endocrine therapy or trastuzumab, chemotherapy remains the backbone of systemic treatment despite its limited efficacy. Available targeted therapies are effective only in a small subset of patients, and in PD-L1–negative, treatment-naïve mTNBC, therapeutic options remain particularly limited [3,32].

Molecular heterogeneity therefore contributes substantially to the variability in treatment response and clinical outcomes observed in TNBC [13,32]. Although several molecular subclassification systems—such as those developed by Lehmann—have improved our biological understanding of TNBC, their clinical implementation has so far been limited.

Hormone receptor positivity thresholds further influence classification and may alter eligibility for specific therapeutic strategies [4,32]. Gene expression analyses have shown that 76% of tumors with low hormone receptor expression (1–9%) are ESR1-negative at the mRNA level, and 48% are classified as basal-like, leading to the proposal of a “triple-negative-like breast cancer” (TN-like BC) category [9,32].

In this context, the lack of validated predictive biomarkers for each subtype remains a major limitation to the clinical applicability of molecular subclassification. Even among tumors with similar gene expression profiles, responses to chemotherapy may differ substantially, as observed in BL1 and BL2 subtypes, which share high Ki-67 expression and enrichment of proliferation-related genes but display distinct therapeutic behaviors. These observations suggest that the clinical value of distinguishing between basal-like and non–basal-like subtypes will not be fully realized until robust and reproducible biomarkers are identified and validated for each subgroup [32,33].

The FUTURE-SUPER trial, previously discussed, provides proof-of-concept that subtype-based therapeutic strategies may partially overcome these limitations by stratifying the TNBC population into four molecular subtypes and assigning tailored treatments accordingly [13]. Unlike earlier gene-centric approaches—where only a small fraction of patients were eligible—this framework offers a broader and more clinically actionable model for precision oncology in TNBC.

## 3. Molecular Subtypes of TNBC: Biology and Clinical Relevance

From a molecular perspective, metastatic triple-negative breast cancer can be subdivided into biologically distinct subtypes defined by specific transcriptomic programs, mutational features, tumor microenvironmental characteristics, and therapeutic vulnerabilities. For descriptive purposes, this section follows the classical Lehmann classification, which includes Basal-like 1 (BL1), Basal-like 2 (BL2), Mesenchymal (M), Mesenchymal Stem-like (MSL), Immunomodulatory (IM), and Luminal Androgen Receptor (LAR) subtypes. This model remains highly informative from a biological standpoint and provides a useful framework for understanding subtype-specific mechanisms of tumor behavior and treatment sensitivity.

However, it is important to emphasize that the classical Lehmann six-subtype model and the clinically oriented Fudan four-subtype system are not identical classifications, but partially overlapping frameworks that capture related biological dimensions. The Lehmann model is particularly useful for describing intrinsic biological programs such as basal-like proliferation, mesenchymal differentiation, immunomodulation, and androgen receptor signaling. In contrast, the Fudan classification—LAR, IM, Basal-like Immune-Suppressed (BLIS), and Mesenchymal-like (MES)—was developed with a more direct translational orientation and has been prospectively incorporated into subtype-guided clinical trials such as FUTURE and FUTURE-SUPER. Broadly, LAR is shared across systems; IM overlaps with immune-enriched tumors; BLIS captures basal-like tumors with immune suppression and high proliferative features; and MES overlaps with mesenchymal and mesenchymal stem-like biology. Nevertheless, these correspondences are approximate rather than interchangeable, and this distinction should be considered when extrapolating biological observations from one classification system to clinical outcomes generated in another.

The main biological and therapeutic features of the major molecular subtypes of mTNBC, together with their approximate correspondence with clinically oriented classification systems, are summarized in Table 1.

### 3.1. Basal-like 1 (BL1)

The key molecular features of the BL1 subtype in metastatic triple-negative breast cancer include marked genomic instability, with a predominance of TP53 mutations (up to 92%) and deletions in genes involved in DNA repair, such as BRCA2, MDM2, PTEN, and RB1. This subtype is characterized by high expression of genes related to cell cycle regulation and DNA damage response, resulting in intense tumor proliferation, elevated Ki-67 levels, and a distinctly aggressive biological phenotype [16,34].

The transcriptomic profile of BL1 is defined by overexpression of genes associated with replication, mitosis, and homologous recombination repair mechanisms. In addition, enrichment of MYC and WNT signaling pathways is observed, together with low expression of immune-related genes, distinguishing this subtype from others such as the Immunomodulatory subtype and, at least in theory, limiting the benefit of immunotherapy [34].

With regard to its mutational landscape, BL1 is the most genomically unstable subtype within TNBC, with a high frequency of TP53 mutations and loss of tumor suppressor genes. The combination of low PTEN expression and high RhoA signaling within this subtype has been associated with particularly poor prognosis (HR = 8.2; *p* = 0.0009) [16].

These features help explain the clinical behavior of the BL1 subtype and its response to available therapies, as it is characterized by greater sensitivity to platinum-based chemotherapy and PARP inhibitors, particularly in the presence of homologous recombination deficiency. This subtype shows some of the highest rates of pathological complete response in the neoadjuvant setting, although survival outcomes may still be influenced by additional alterations such as PTEN loss [9,16,34].

From a therapeutic standpoint, the most relevant implications include prioritizing the use of DNA-damaging agents such as platinum compounds and considering PARP inhibitors in patients with BRCA1/2 alterations or homologous recombination repair deficiency. The identification of additional biomarkers, such as PTEN and RhoA, may contribute to more accurate prognostic stratification and more refined therapeutic selection.

### 3.2. Basal-like 2 (BL2)

The BL2 subtype displays distinctive molecular features within the spectrum of metastatic TNBC. It is characterized by high expression of genes related to growth factors (EGF, MET, NGF), glutathione metabolism, and E2F2, TGFβ, and CXCL8 signaling. Its transcriptomic profile is distinguished by activation of receptor tyrosine kinase-dependent pathways and cellular proliferation programs, with overexpression of genes such as FGFR4, ERBB4, and other tyrosine kinases, thereby differentiating it from the BL1 subtype. In addition, it is usually associated with a less immune-reactive tumor microenvironment and a greater stromal component [35].

From a mutational standpoint, BL2 is characterized by copy number gain and high AKT1 expression, an alteration associated with worse prognosis (HR = 3.9; *p* = 0.02), as well as mutations in genes involved in survival and proliferation pathways. Compared with BL1, it harbors a lower frequency of BRCA1/2 mutations, but greater activation of growth signaling pathways and increased resistance to chemotherapy [34,35,36].

Taken together, these features translate into a less favorable clinical behavior, with lower response rates to neoadjuvant chemotherapy and shorter relapse-free survival than that observed in BL1. Patients with BL2 therefore tend to have a poorer prognosis and a higher risk of early relapse [36].

Therapeutically, the BL2 subtype supports investigation of tyrosine kinase inhibitors targeting FGFR, ERBB4, or MET, as well as PI3K/AKT pathway inhibitors. The high expression of AKT1 and other receptor tyrosine kinases suggests that targeted therapy may be more effective than standard chemotherapy in this subtype, although no formal recommendation has yet been incorporated into international guidelines [34,35,36].

### 3.3. Mesenchymal (M)

The molecular features of the M subtype in metastatic triple-negative breast cancer include high expression of genes associated with epithelial–mesenchymal transition (EMT), extracellular matrix remodeling, and growth factor signaling pathways such as PDGF and c-Kit. This subtype is distinguished by prominent stromal signatures, cancer-associated fibroblasts, and angiogenesis-related genes, together with a tumor microenvironment poor in immune cells and low PD-L1 expression [37].

At the transcriptomic level, the M subtype shows overexpression of EMT markers, matrix metalloproteinases, and genes related to cell motility and invasion. In parallel, transcriptional repression of genes involved in antigen presentation, particularly MHC-I, is observed, mediated by H3K27me3 modifications through the PRC2 repressive complex, which substantially contributes to immune evasion [37,38].

Beyond transcriptional programs, the mesenchymal phenotype is also shaped by the physical properties of tumor cells and the extracellular matrix. M and MSL tumors frequently display increased matrix deposition, collagen remodeling, altered integrin signaling, and enhanced focal adhesion dynamics. These changes can modify cell stiffness, adhesion forces, mechanotransduction, and invasive capacity, thereby facilitating migration through dense stromal environments and promoting resistance to cytotoxic therapies. Activation of pathways such as FAK/SRC, RhoA/ROCK, YAP/TAZ, and TGFβ signaling may link extracellular matrix rigidity to EMT, stemness, immune exclusion, and therapeutic resistance. Therefore, the biology of mesenchymal-like TNBC should not be interpreted only as a gene expression phenomenon, but also as a biomechanical state in which tumor cells and stromal components cooperate to generate an invasive and treatment-resistant niche [39].

With regard to the mutational landscape, the M subtype exhibits a high mutational burden and marked genomic instability, although without a single dominant driver alteration. Diverse abnormalities are observed in the RTK-RAS pathway, along with frequent copy number losses in regions such as 5q and 15q, which include genes involved in immune functions [38].

These features explain its clinical behavior, which is characterized by poor sensitivity to standard chemotherapy and a particularly invasive and aggressive phenotype. M tumors show lower pathological complete response rates and worse prognosis than basal-like and immunomodulatory subtypes [37,38].

From a therapeutic standpoint, this subtype may be sensitive to inhibitors targeting the RTK-RAS and PI3K/mTOR pathways. In addition, pharmacologic inhibition of PRC2 (EZH2/EED) may restore MHC-I expression and enhance chemotherapy efficacy in preclinical models, supporting the rationale for exploring PRC2 inhibitors in PD-L1-negative mesenchymal tumors. Given its immunologically “cold” microenvironment, immunotherapy is unlikely to be effective as monotherapy, and combination strategies targeting the stroma and metabolic vulnerabilities are currently under investigation [37,38].

### 3.4. Mesenchymal Stem-like (MSL)

The molecular features of the MSL subtype include prominent expression of genes associated with mesenchymal stem cells, epithelial–mesenchymal transition (EMT), and growth factor signaling pathways. This subtype was originally identified by Lehmann and colleagues as one of the six molecular TNBC subtypes, although subsequent studies suggested that much of its transcriptomic signature may derive from tumor-associated stromal cells rather than from the tumor cells themselves. It is therefore currently regarded more as a microenvironment-influenced variant than as a purely intrinsic tumor subtype. The MSL subtype is distinguished by strong EGFR signaling and expression of genes related to stem-like properties, including markers such as CD44 and ALDH1A1 [40].

Its transcriptomic profile is characterized by overexpression of genes involved in EMT, stromal interaction, and cell motility, as well as pathways mediated by growth factors such as PDGF, c-Kit, and EGFR [41]. Both the M and MSL subtypes show high angiogenesis signature scores, suggesting greater dependence on tumor neovascularization and, potentially, greater sensitivity to antiangiogenic agents such as bevacizumab. Laser-capture microdissection analyses have demonstrated that many of the transcripts characteristic of the MSL subtype predominantly originate from tumor-associated stromal cells, particularly fibroblasts, further reinforcing doubts as to whether it represents a truly intrinsic tumor subtype [42].

From a mutational standpoint, MSL harbors alterations in growth signaling pathways, with enrichment in the RTK-RAS pathway and possible sensitivity to RTK inhibitors. Unlike the BL1 subtype, MSL displays a moderate degree of genomic instability and mutational burden. The Wnt/β-catenin and Wnt/PCP pathways appear to be particularly relevant in the acquisition of EMT, stemness, and tumor stem cell properties in this context [43].

Clinically, the MSL subtype has been associated with younger age at diagnosis, metaplastic or mesenchymal histopathologic features, and a prominent stromal component [40,41]. Available studies report lower pathological complete response rates than in BL1 and intermediate responses to standard neoadjuvant chemotherapy [42,43]. It has also been associated with shorter overall survival, with median survival of 68.2 months reported in immunohistochemistry-based subclassification studies [44].

From a therapeutic perspective, the MSL subtype may show sensitivity to PI3K/mTOR inhibitors (NVP-BEZ235) and dasatinib (an ABL/SRC inhibitor) in representative cellular models. Its association with high angiogenesis signatures supports a potential role for antiangiogenic agents such as bevacizumab; notably, the FUTURE-SUPER trial demonstrated benefit with bevacizumab combined with nab-paclitaxel in BLIS/MES subtypes without PI3K/AKT mutations (median PFS 9.4 vs. 3.9 months; HR 0.41) [13,45,46]. In addition, the combination of CHK1 and BCL2 inhibitors has shown selective efficacy in preclinical models of MSL breast cancer. The immunologically “cold” nature of this subtype is likely to limit the efficacy of immunotherapy as monotherapy, although strategies aimed at reversing EMT and restoring tumor immunogenicity remain under investigation [45,46].

### 3.5. Immunomodulatory (IM)

The molecular features of the IM subtype include high expression of genes related to the adaptive immune response, such as PD-1, PD-L1, and CTLA-4, together with robust infiltration by FOXP3+ regulatory T cells and CD8+ cells within the tumor microenvironment. This subtype is associated with high expression of immune-related signatures and immune checkpoint genes, along with activation of the Notch pathway and enrichment in interferon-α and interferon-γ signaling. From a transcriptomic standpoint, IM is characterized by overexpression of genes involved in lymphocyte activation, antigen presentation, and immune response regulation. It displays an inflammatory profile dominated by adaptive T cells and high TIL density, clearly distinguishing it from immunologically “cold” subtypes [47].

The mutational landscape of the IM subtype includes specific alterations in genes such as CTNNB1 (β-catenin) and IDH1, as well as a higher frequency of PD-L1 expression (≥1%) and activation of the Notch pathway. It is not a subtype characterized by particularly high overall mutational burden, but rather by differential regulation of lincRNAs associated with better prognosis [47,48].

Clinically, the IM subtype is associated with a better prognosis than other TNBC subtypes, longer progression-free survival, and greater sensitivity to immunotherapy. However, very high PD-1 expression within this subtype may paradoxically be associated with poorer prognosis [46,47,48].

The therapeutic implications of the IM subtype are particularly relevant, as it shows marked sensitivity to immune checkpoint inhibitors—such as pembrolizumab, atezolizumab, or camrelizumab—especially in PD-L1-positive tumors with abundant CD8+ infiltration. The FUTURE-SUPER trial demonstrated that the combination of chemotherapy, antiangiogenic therapy, and immunotherapy (famitinib, camrelizumab, and nab-paclitaxel) prolonged progression-free survival in the IM subtype (median 15.1 months; HR 0.46) [13]. Optimal patient selection for immunotherapy in this setting should take into account multiple biomarkers, including PD-L1 and CD8+ infiltration [46,47,48].

### 3.6. Luminal Androgen Receptor (LAR)

The key molecular features of the Luminal Androgen Receptor (LAR) subtype in metastatic triple-negative breast cancer include high expression of the androgen receptor (AR) and AR-dependent target genes, together with a luminal profile and absence of basal markers such as CK5/6 and p63. LAR accounts for approximately 10–16% of TNBC cases and is associated with high positivity for AR, FOXA1, and FGFR4, low proliferative activity (low Ki-67), and less aggressive histologic features compared with other subtypes [48].

From a transcriptomic standpoint, LAR shows overexpression of genes related to AR signaling, FOXA1, and ERBB2, together with low expression of proliferation-associated genes, CDK6, BRCAness-related signatures, and p53. In addition, enrichment of interferon, JAK-STAT, and androgen signaling pathways is observed, particularly in AR-positive epithelial cells [48,49].

The mutational landscape of LAR is characterized by a high frequency of mutations in PIK3CA (up to 55%), AKT1 (13%), and CDH1 (13%), as well as deletions in CD274 (PD-L1) and PDCD1LG2 (PD-L2), and a low homologous recombination deficiency (HRD) score. AR splice variants (AR-SV) have also been identified in up to 20% of cases and are associated with more aggressive phenotypes [49].

Clinically, LAR is associated with lower sensitivity to standard chemotherapy and with the lowest pathological complete response rate among TNBC subtypes. It also shows a tendency toward bone and nodal metastases and has been linked to worse overall prognosis (HR = 1.47; 95% CI: 1.0–2.14) [16,48,49,50,51,52]. However, some studies suggest that it may also be associated with lower histologic aggressiveness and a lower proliferative index [48,49].

These features support the potential clinical benefit of antiandrogen strategies such as enzalutamide or darolutamide—approved for prostate cancer but not for TNBC—with modest activity in AR-positive TNBC. Enzalutamide has shown clinical benefit rates of approximately 25% in patients selected by AR immunohistochemistry, whereas darolutamide achieved a 16-week clinical benefit rate of 29% in the UCBG 3-06 START trial, with greater benefit in molecular apocrine-high tumors. These findings suggest that optimal selection likely requires transcriptomic evidence of high androgen pathway activity rather than AR immunohistochemistry alone [50,51,52,53]. LAR tumors harboring PIK3CA or AKT1 mutations may derive additional benefit from PI3K/mTOR pathway inhibitors, particularly in cases resistant to antiandrogens, although this evidence remains largely preclinical and these agents are currently approved mainly in luminal tumors [54]. In this context, combining antiandrogens with PI3K or CDK4/6 inhibitors represents a promising strategy under clinical evaluation, whereas FGFR4 inhibition remains more exploratory [55].

Overall, molecular subclassification of TNBC has transformed our understanding of this heterogeneous disease, demonstrating that each subtype (BL1, BL2, M, MSL, IM, and LAR) possesses distinct biological, genomic, and clinical characteristics that shape both prognosis and therapeutic response. The BL1 subtype appears to be the most sensitive to chemotherapy and platinum-based regimens, the IM subtype shows particular susceptibility to immunotherapy, and the LAR subtype represents a clear candidate for antiandrogen-based strategies and PI3K inhibition. The integration of an appropriate molecular classification system could enable more accurate patient stratification and a more rational selection of targeted therapies, with the potential to significantly improve outcomes in this population.

## 4. Subtype-Guided Therapeutic Strategies in Metastatic TNBC

### 4.1. Chemotherapy Sensitivity by Subtype

Chemotherapy sensitivity in metastatic triple-negative breast cancer varies substantially according to molecular subtype and it has to be noted that most of these data derive primarily from neoadjuvant settings but provide a useful biological framework for understanding differential chemosensitivity in metastatic disease. The Basal-like 1 (BL1) subtype shows the highest pathological complete response (pCR) rate, reaching up to 65.6% with carboplatin- and docetaxel-based regimens; Basal-like 2 (BL2) shows an intermediate rate (47.4%), whereas the Mesenchymal (M) and Luminal Androgen Receptor (LAR) subtypes have clearly lower rates (36.4% and 21.4%, respectively) [56,57]. These findings have been validated in independent cohorts and confirm that BL1 is the most chemosensitive subtype, particularly to DNA-damaging agents such as platinum compounds [56].

This differential sensitivity can be explained by the molecular biology of each subtype. BL1 and BL2 tumors display a high proliferative rate and a greater likelihood of homologous recombination deficiency, which enhances the activity of cytotoxic chemotherapy and platinum agents and supports the observation that BL1 appears to be the most chemosensitive subtype. By contrast, the M and LAR subtypes show lower proliferation, greater biological plasticity, and a higher degree of relative resistance [34,37,38,56,58]. In addition, the presence of BRCA1/2 mutations and a BRCA-like phenotype is associated with increased sensitivity to platinum agents and PARP inhibitors [56,57].

Chemoresistance in mTNBC is not explained solely by proliferation rate or HRD status. Resistance to anthracyclines may arise through enhanced DNA repair capacity, altered topoisomerase IIα expression or function, increased antioxidant defenses, drug efflux mediated by ATP-binding cassette transporters, and impaired apoptosis through dysregulation of p53, BCL-2 family proteins, or PI3K/AKT signaling. In tumors with partial preservation or restoration of homologous recombination repair, the cytotoxic effect of DNA-damaging agents may be attenuated, limiting the durability of response even in initially sensitive basal-like tumors.

Taxane resistance is also multifactorial and may involve altered microtubule dynamics, differential expression of β-tubulin isoforms, activation of survival pathways such as PI3K/AKT/mTOR and MAPK, epithelial–mesenchymal transition, cancer stem-like enrichment, and stromal-mediated drug tolerance. Mesenchymal-like tumors may be particularly resistant because EMT-associated plasticity and extracellular matrix remodeling can reduce drug-induced apoptosis, promote survival under cellular stress, and generate protective tumor microenvironmental niches. In addition, immune-suppressive macrophages, cancer-associated fibroblasts, hypoxia, and abnormal vasculature may reduce effective drug delivery and promote adaptive resistance. Therefore, chemotherapy sensitivity by subtype should be interpreted as the result of an integrated biological state combining proliferation, DNA repair deficiency, apoptotic competence, stromal architecture, immune contexture, and drug transport mechanisms [58].

**Figure 2:**
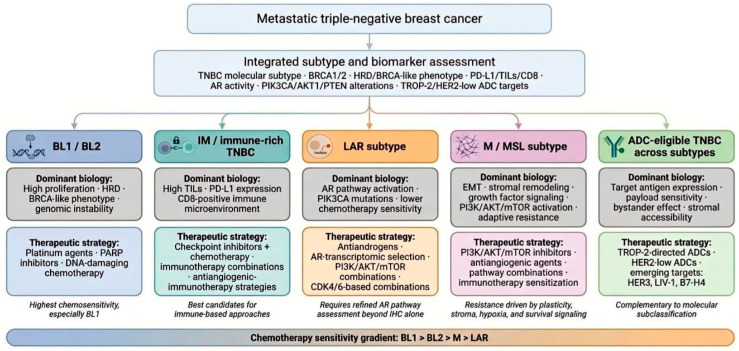
Subtype-guided therapeutic strategies in metastatic triple-negative breast cancer. This figure illustrates a conceptual framework for integrating molecular subtype, validated biomarkers, tumor microenvironmental features, prior treatment exposure, and therapeutic targets into treatment selection for metastatic triple-negative breast cancer (mTNBC). Immune-enriched or immunomodulatory tumors with PD-L1 expression, high tumor-infiltrating lymphocytes (TILs), and CD8+ infiltration may derive benefit from immunotherapy-based strategies. Basal-like or homologous recombination deficiency (HRD)-enriched tumors may be more suitable for platinum-based chemotherapy, poly(ADP-ribose) polymerase (PARP) inhibitors in BRCA1/2-mutated or HRD-selected disease, and DNA damage response-targeted approaches. Luminal androgen receptor (LAR) tumors may represent candidates for androgen receptor-directed strategies, particularly when androgen receptor pathway activity is confirmed, and for combinations targeting PI3K/AKT/mTOR or cell-cycle pathways. Mesenchymal and mesenchymal stem-like tumors are characterized by epithelial–mesenchymal transition (EMT), stromal activation, extracellular matrix remodeling, angiogenesis, and adaptive resistance, supporting investigation of PI3K/mTOR, antiangiogenic, stromal-targeting, and clinical trial-based strategies. Antibody–drug conjugates (ADCs), including TROP-2-directed and HER2-low-directed approaches, represent important cross-subtype therapeutic options. This algorithm is intended as a biologically informed conceptual model and should complement, rather than replace, guideline-based clinical decision-making. Abbreviations: ADC, antibody–drug conjugate; AR, androgen receptor; EMT, epithelial–mesenchymal transition; HER2, human epidermal growth factor receptor 2; HRD, homologous recombination deficiency; IM, Immunomodulatory; LAR, Luminal Androgen Receptor; mTNBC, metastatic triple-negative breast cancer; MSL, Mesenchymal Stem-like; PARP, poly(ADP-ribose) polymerase; TILs, tumor-infiltrating lymphocytes.

Taken together, these data support the notion that chemosensitivity is not uniform across TNBC. BL1 represents the subtype with the highest likelihood of response to conventional chemotherapy, followed by BL2, whereas the M and LAR subtypes derive clearly more limited benefit, further underscoring the need for treatment strategies tailored to the molecular profile. Beyond its biological relevance, molecular subclassification may also provide a clinically useful framework for therapeutic stratification in metastatic TNBC which is summarized in Figure 2.

### 4.2. Role of PARP Inhibitors and Platinum Agents (BL1/BL2)

The role of poly(ADP-ribose) polymerase (PARP) inhibitors and platinum agents is particularly relevant in basal-like TNBC, especially BL1 tumors, because this subtype is enriched for proliferative, cell-cycle and DNA-damage response programs and may overlap with germline or somatic BRCA1/2 alterations, homologous recombination deficiency (HRD), and BRCA-like biology [14,37,42,56,59,60].

In patients with germline BRCA1/2-mutated, HER2-negative advanced breast cancer, PARP inhibitors such as olaparib and talazoparib have demonstrated improved progression-free survival compared with physician’s choice chemotherapy and are approved for this molecularly defined population. In parallel, platinum compounds have shown particular activity in germline BRCA1/2-mutated disease. In the TNT trial, carboplatin achieved a significantly higher objective response rate than docetaxel in patients with germline BRCA1/2-mutated breast cancer, reaching 68.0% versus 33.3%, whereas this advantage was not observed in the unselected TNBC population [60,61,62,63,64].

Beyond germline BRCA1/2 mutations, BRCA-like biology may also identify tumors sensitive to DNA-damaging strategies. In the randomized phase II S1416 trial, the addition of veliparib to cisplatin significantly improved progression-free survival in patients with BRCA-like metastatic TNBC compared with cisplatin alone, with median PFS of 5.9 versus 4.2 months, respectively (HR 0.57; *p* = 0.010). This benefit was observed in the predefined BRCA-like subgroup rather than in non-BRCA-like tumors [59]. Although the combination of PARP inhibitors and platinum agents may increase hematologic toxicity, this toxicity is generally manageable with dose modifications and appropriate supportive care.

Overall, PARP inhibitors and platinum agents represent clinically relevant options for metastatic TNBC with germline BRCA1/2 mutations or BRCA-like/HRD features. Their relevance is particularly strong in basal-like/BL1 tumors, but treatment selection should be based on validated biomarkers of DNA repair deficiency rather than molecular subtype alone [57,59,60,61,62,63,64].

### 4.3. Immunotherapy in IM and Immune-Rich Phenotypes

Recent immunotherapy data in TNBC support a clear role for immune checkpoint blockade in the perioperative treatment of high-risk early-stage disease and in first-line PD-L1-positive metastatic disease, whereas purely adjuvant immunotherapy after surgery and chemotherapy has shown more limited benefit.

In high-risk early-stage TNBC, pembrolizumab in combination with neoadjuvant chemotherapy, followed by adjuvant pembrolizumab, has significantly improved both event-free survival and overall survival. In the phase III KEYNOTE-522 trial, the addition of pembrolizumab produced an absolute 4.9 percentage point improvement in 5-year overall survival and an approximately 35% relative reduction in the risk of progression, recurrence, or death, together with a reduction in distant recurrences. This benefit was observed regardless of PD-L1 status and was generally consistent across clinically relevant subgroups defined by tumor size and nodal status [65]. By contrast, purely adjuvant atezolizumab added to postoperative chemotherapy did not improve invasive disease-free survival in ALEXANDRA/IMpassion030 and is not recommended in this setting [66,67].

In metastatic disease, immune checkpoint inhibitors combined with chemotherapy have become a key therapeutic strategy for selected patients with PD-L1-positive advanced TNBC, whereas checkpoint inhibitor monotherapy has limited activity in unselected mTNBC. Pembrolizumab-based chemoimmunotherapy is currently the most clinically relevant approach in this setting, while the role of atezolizumab has become more restricted after heterogeneous results across trials and regulatory changes in some jurisdictions. Overall, benefit from immunotherapy appears enriched in tumors with strong immune activation, high PD-L1 expression, abundant tumor-infiltrating lymphocytes, or tumor-agnostic biomarkers such as high tumor mutational burden [68,69,70,71,72,73].

From a molecular-subtype perspective, immunotherapy is particularly relevant in immune-enriched TNBC phenotypes. Although the original Immunomodulatory subtype largely reflects immune and stromal components rather than a purely tumor-intrinsic program, immune-rich tumors are characterized by higher tumor-infiltrating lymphocyte density, immune-checkpoint expression, antigen-presentation signatures and greater likelihood of response to immune checkpoint blockade [38,47,48,70,71,72].

The FUTURE-SUPER trial further supports this concept in metastatic TNBC. In this subtype-guided first-line phase II trial, patients assigned to the IM cohort, defined by CD8 positivity, derived substantial benefit from nab-paclitaxel combined with camrelizumab and famitinib, achieving a median progression-free survival of 15.1 months and a marked reduction in the risk of progression compared with nab-paclitaxel alone [13]. These data suggest that biomarker selection using CD8 expression, TILs and immune-enrichment features, in addition to PD-L1, may improve the identification of patients most likely to benefit from immunotherapy-based combinations.

This differential sensitivity appears to be driven by an immune-enriched tumor microenvironment that facilitates responsiveness to checkpoint blockade. In contrast, immune-suppressed phenotypes such as BLIS appear to derive more limited benefit from immunotherapy-based approaches [18]. Likewise, combining immunotherapy with chemotherapy and antiangiogenic agents may further enhance efficacy by promoting vascular normalization, reducing immunosuppressive myeloid signaling and improving immune-cell infiltration, although this strategy remains dependent on adequate biomarker selection and further validation [13,74].

Overall, immunotherapy currently represents the most rational strategy for immune-enriched metastatic TNBC, particularly when incorporated into combination regimens and guided by appropriate immunologic biomarker selection rather than by anatomical subtype classification alone [75].

### 4.4. Anti-Androgens and Combination Strategies in LAR

Antiandrogen therapy represents one of the most promising targeted strategies for the Luminal Androgen Receptor (LAR) subtype of metastatic triple-negative breast cancer, which is characterized by high androgen receptor (AR) expression, luminal/apocrine transcriptional programs, and frequent activation of the PI3K/AKT pathway. Clinical trials with AR antagonists such as bicalutamide, enzalutamide and darolutamide have shown modest activity when patients are selected solely on the basis of AR immunohistochemistry, with 16-week clinical benefit rates generally ranging from approximately 19% to 29%. However, benefit appears substantially enriched when selection is refined by transcriptomic evidence of androgen pathway activation, as illustrated by the UCBG 3-06 START trial, in which darolutamide achieved a 16-week clinical benefit rate of 57% in molecular apocrine-high tumors compared with 16% in molecular apocrine-low tumors [53,76].

The phase II UCBG 3-06 START trial evaluated darolutamide versus capecitabine in advanced AR-positive TNBC. Although darolutamide did not meet the prespecified clinical benefit endpoint in the overall AR-positive population, biomarker analysis showed that molecular apocrine-high tumors derived substantially greater benefit than molecular apocrine-low tumors. This finding highlights that AR expression by immunohistochemistry alone is insufficient to identify truly androgen-dependent tumors and that transcriptomic characterization may enable more precise patient selection [53].

The MDV3100-11 phase II trial of enzalutamide in 118 patients with AR-positive TNBC reported a 16-week clinical benefit rate of 25% in the intention-to-treat population and 33% in the evaluable subgroup, defined by AR expression ≥10% by immunohistochemistry. Median progression-free survival was 2.9 months in the intention-to-treat population and 3.3 months in the evaluable subgroup, while median overall survival reached 17.6 months in evaluable patients. Responses were more favorable in patients whose tumors were positive for an AR-related gene-expression signature [76].

To enhance the efficacy of antiandrogen therapy, rational combination strategies are being explored. The TBCRC 032 trial investigated enzalutamide alone or in combination with taselisib, a PI3K inhibitor, in AR-positive metastatic TNBC. The combination increased the clinical benefit rate to 35.7%, and exploratory analyses suggested that patients with LAR tumors had higher clinical benefit than non-LAR patients, with a trend toward improved response and longer progression-free survival. Genomic analyses further revealed subtype-specific response patterns, including FGFR2 fusions and AR splice variants, supporting the need for molecular refinement beyond AR immunohistochemistry alone [77].

Promising preclinical data have also been reported with the combination of enzalutamide and HDAC inhibitors such as chidamide, which demonstrated synergistic activity in preclinical models and patient-derived TNBC-LAR organoids by interfering with tumor metabolism and autophagy [78]. Similarly, combined inhibition of CDK4/6 and AKT, for example with palbociclib plus capivasertib, has shown significant synergy in LAR cellular models and more potent inhibition of patient-derived xenograft growth than some alternative combinations. The lack of enzalutamide activity in selected cell lines suggests that, in certain LAR models with limited AR dependence, direct AKT pathway inhibition may be more relevant than AR blockade [79].

In enzalutamide-resistant TNBC-LAR models, mTOR and PI3K inhibitors have demonstrated antitumor activity in vivo in xenografts harboring PIK3CA and AKT1 alterations, supporting the hypothesis that resistance to antiandrogen therapy may be mediated by compensatory activation of the PI3K/AKT/mTOR pathway [54]. This observation is particularly relevant because PIK3CA mutations are enriched in AR-positive TNBC, with early studies reporting frequencies close to 40% in AR-positive tumors compared with approximately 4% in AR-negative tumors [80].

The randomized phase II TBCRC 058 trial (NCT06099769) is currently evaluating enzalutamide, enzalutamide plus mifepristone, a glucocorticoid receptor antagonist, and physician’s choice chemotherapy in AR-positive metastatic TNBC and ER-low metastatic breast cancer, with the aim of determining whether glucocorticoid receptor inhibition can overcome mechanisms of resistance to antiandrogen therapy [81,82].

Other combinations under development include enzalutamide with CDK7 inhibitors such as KRLS-017, which has shown synergistic antiproliferative effects in AR-positive TNBC models through inhibition of c-MYC-driven tumorigenesis [83]. Additional strategies targeting HER-family signaling or downstream PI3K/AKT/mTOR activation remain biologically plausible in selected LAR tumors, but require further clinical validation [81,82,83,84].

Taken together, antiandrogen therapy and rational combination strategies—particularly those guided by AR transcriptomic activity and by co-targeting PI3K/AKT/mTOR signaling or cell-cycle regulators—represent one of the most promising molecularly informed approaches for the LAR subtype, which generally responds poorly to conventional chemotherapy. Optimal patient selection, however, requires functional assessment of AR pathway activity rather than immunohistochemical detection alone, together with identification of concurrent PIK3CA/AKT1 alterations to support biologically informed combination strategies.

### 4.5. Targeting PI3K/AKT/mTOR and Growth Pathways in M/MSL

The PI3K/AKT/mTOR pathway is dysregulated in a substantial proportion of triple-negative breast cancers, making it a biologically relevant therapeutic target, particularly in mesenchymal-like phenotypes enriched for growth factor-mediated signaling and in LAR tumors with frequent PI3K pathway alterations [84,85,86].

#### 4.5.1. mTOR Inhibitors

The mTOR inhibitor everolimus has been evaluated in several clinical trials in TNBC. A single-arm phase II study combining everolimus with carboplatin in metastatic TNBC reported a clinical benefit rate of 36%, including 1 complete response, 6 partial responses and 7 cases of stable disease, with a median progression-free survival of 3 months and a median overall survival of 16.6 months [87]. In the neoadjuvant setting, a randomized phase II trial of cisplatin and paclitaxel with or without everolimus in stage II/III TNBC did not demonstrate an improvement in pathological complete response with the addition of everolimus, with pCR rates of 36% versus 49%, respectively. Exploratory correlative analyses suggested that TNBC molecular subtype, AR status, Ki67 and DNA-damage response alterations may influence clinical response and long-term outcome [88].

In preclinical patient-derived xenograft models, everolimus inhibited tumor growth in 7 of 15 models, including tumors with basal-like, LAR, mesenchymal and HER2-enriched transcriptional features, suggesting that response to everolimus is not restricted to a single TNBC subtype. Interestingly, response correlated with treatment-induced increases in AKT phosphorylation, whereas baseline markers of PI3K pathway activation were insufficient to predict sensitivity [89].

#### 4.5.2. AKT Inhibitors

AKT inhibitors such as ipatasertib and capivasertib have shown signals of activity in phase II trials, although phase III results have been inconsistent. The phase II LOTUS trial demonstrated that ipatasertib plus paclitaxel improved progression-free survival compared with placebo plus paclitaxel, with median PFS of 6.2 versus 4.9 months, respectively (HR 0.60; *p* = 0.037). Benefit appeared more pronounced in tumors harboring PIK3CA/AKT1/PTEN alterations, with median PFS of 9.3 versus 3.7 months (HR 0.30) [90,91]. However, the phase III IPATunity130 trial failed to confirm this benefit in a biomarker-selected population with PIK3CA/AKT1/PTEN-altered tumors, reporting median PFS of 7.4 versus 6.1 months (HR 1.02), underscoring the complexity of biological selection in this setting [92].

The phase II PAKT trial evaluated capivasertib plus paclitaxel versus placebo plus paclitaxel as first-line treatment for metastatic TNBC and demonstrated significant improvements in both progression-free survival, with median PFS of 5.9 versus 4.2 months (HR 0.74), and overall survival, with median OS of 19.1 versus 12.6 months (HR 0.61). In patients with tumors harboring PIK3CA/AKT1/PTEN alterations, median PFS was 9.3 versus 3.7 months (HR 0.30; *p* = 0.01) [93]. Nevertheless, the phase III CAPItello-290 trial did not meet its primary endpoint of improving overall survival, with median OS of 17.7 versus 18.0 months (HR 0.92; *p* = 0.32). Although progression-free survival numerically favored capivasertib plus paclitaxel, particularly in tumors harboring PIK3CA/AKT1/PTEN alterations, this signal did not translate into a statistically significant overall survival advantage [94].

A recent meta-analysis of randomized trials in advanced or metastatic breast cancer suggested that AKT inhibitors may improve survival outcomes, particularly in tumors harboring PIK3CA/AKT1/PTEN alterations. However, because this evidence is not restricted to TNBC and includes biologically heterogeneous breast cancer populations, its applicability to metastatic TNBC should be interpreted cautiously [95].

#### 4.5.3. Dual PI3K–mTOR Inhibition

Dual inhibition of PI3K and mTOR with paxalisib has shown, in preclinical TNBC models, the capacity to reduce tumor growth, migration, metastatic dissemination and resistance to immunotherapy. Mechanistically, this effect appears to involve modulation of EZH2-dependent programs, including the repressive p85β–EZH2–H3K27me3 axis and the active EZH2–NFκB axis, thereby promoting a less mesenchymal and more immune-visible phenotype and potentially enhancing sensitivity to immunotherapy in resistant tumors [96].

#### 4.5.4. Antiangiogenic Agents

Mesenchymal-like TNBC phenotypes have been associated with high angiogenesis signatures, supporting the rationale for antiangiogenic strategies. In FUTURE-SUPER, patients in the BLIS/MES-PI3K/AKT wild-type cohort received nab-paclitaxel plus bevacizumab and achieved longer progression-free survival than those treated with nab-paclitaxel alone, supporting antiangiogenic therapy as a rational component of subtype-guided treatment in this biologically defined subgroup [13].

In the neoadjuvant setting, the GeparQuinto trial showed that the addition of bevacizumab to chemotherapy significantly increased the pCR rate in TNBC, from 27.9% to 39.3% (*p* = 0.003), with the clearest benefit observed in the triple-negative subgroup [97]. A meta-analysis of seven studies including 5408 patients confirmed that bevacizumab improves pCR rates in TNBC (OR 1.55; 95% CI: 1.29–1.86; *p* < 0.00001), although pCR improvements have not consistently translated into survival benefit, limiting routine use in early TNBC [98].

#### 4.5.5. Combination Strategies

Given the complexity of resistance mechanisms in M and MSL phenotypes, combination strategies may be necessary to optimize therapeutic efficacy. In preclinical TNBC models, CDK8/19 inhibitors have shown synergy with everolimus and capivasertib and potentiated tumor suppression in vivo by counteracting the transcriptional adaptation that limits the efficacy of PI3K/AKT/mTOR pathway inhibitors [99].

The combination of PI3K inhibitors with CDK4/6 inhibitors, such as alpelisib and ribociclib, has also shown preclinical synergy in TNBC, with greater suppression of p-S6, reduction in MCL-1, induction of apoptosis and stronger inhibition of tumor growth in PDX models [100]. Likewise, the combination of everolimus with gefitinib induced synergistic growth inhibition in TNBC cell lines harboring activating PI3K mutations, associated with simultaneous inhibition of mTOR and P70S6K phosphorylation [101].

In the immunologic setting, the combination of everolimus with anti-PD-1 therapy normalized tumor vasculature, restored CD8+ T-cell infiltration and reduced tumor growth in TNBC models, overcoming the resistance observed with everolimus alone [102]. In addition, a phase I/II trial is evaluating eribulin plus copanlisib in metastatic TNBC, supported by preclinical data suggesting enhanced antitumor activity irrespective of PIK3CA/PTEN mutational status, although clinical efficacy data remain pending [103].

Overall, the PI3K/AKT/mTOR pathway represents a biologically relevant therapeutic target in mesenchymal-like TNBC phenotypes, although the efficacy of single-agent inhibitors has been limited by adaptive resistance mechanisms. Combination strategies integrating pathway inhibitors with antiangiogenic agents, cell-cycle inhibitors or immunotherapy, together with appropriate biomarker-based selection, remain an active area of preclinical and early clinical investigation.

### 4.6. Antibody–Drug Conjugates Across Molecular Subtypes

Antibody–drug conjugates (ADCs) have become an essential component of the therapeutic landscape of metastatic TNBC and represent a distinct form of precision oncology. Unlike transcriptomic subtype-directed therapies, ADC activity is primarily determined by target antigen expression, antibody internalization, linker stability, payload potency, drug-to-antibody ratio, bystander effect, and tumor microenvironmental factors. Therefore, ADCs may be clinically active across multiple TNBC molecular subtypes, even when no dominant oncogenic driver is identified.

Sacituzumab govitecan, a TROP-2-directed ADC carrying the topoisomerase I inhibitor SN-38, has demonstrated clinically meaningful activity in metastatic TNBC and represents one of the most relevant therapeutic options after prior systemic therapy. In the phase III ASCENT trial, sacituzumab govitecan improved progression-free survival and overall survival compared with single-agent chemotherapy in patients with relapsed or refractory metastatic TNBC, establishing TROP-2-directed ADC therapy as a major advance in this disease [104]. Because TROP-2 is broadly expressed across epithelial breast cancers, the activity of sacituzumab govitecan is not restricted to a single molecular subtype. Nevertheless, subtype biology may influence response through differences in proliferation, DNA damage response, drug efflux, immune contexture, and stromal accessibility. Basal-like tumors may be particularly susceptible to topoisomerase I inhibitor payloads because of high proliferative activity and genomic instability, whereas mesenchymal-like tumors may display relative resistance due to stromal barriers, EMT-associated drug tolerance, and altered apoptotic signaling.

Trastuzumab deruxtecan, a HER2-directed ADC with a topoisomerase I inhibitor payload and strong bystander effect, has expanded the therapeutic relevance of HER2 expression beyond classical HER2-positive disease. In DESTINY-Breast04, trastuzumab deruxtecan significantly improved progression-free survival and overall survival compared with physician’s choice chemotherapy in patients with HER2-low metastatic breast cancer, defined as HER2 immunohistochemistry 1+ or 2+ with negative in situ hybridization [105]. This is particularly relevant for TNBC because a subset of tumors classified as HER2-negative by conventional criteria may fall into the HER2-low category and therefore become candidates for HER2-low-directed ADC strategies. The clinical relevance of trastuzumab deruxtecan in HER2-low disease illustrates the limitations of rigid receptor-based classification and supports a more biomarker-driven approach.

From a subtype perspective, ADCs should be viewed as complementary rather than competitive with molecular subclassification. Molecular subtyping may help identify biological contexts of sensitivity or resistance, whereas ADC target assessment determines treatment eligibility. Future studies should evaluate whether TROP-2, HER2-low status, HER3, LIV-1, B7-H4, or other ADC targets vary according to TNBC subtype and whether transcriptomic or immune features can improve prediction of ADC benefit. In this framework, ADCs may function as broadly active therapeutic platforms that can be further refined by integrating antigen expression, payload sensitivity, DNA damage repair capacity, and tumor microenvironmental architecture [106].

## 5. Clinical Trials Incorporating Molecular Subtyping in mTNBC: Clinical Evidence and Ongoing Development

The prospective incorporation of molecular subclassification into clinical development in metastatic triple-negative breast cancer addresses a clear unmet need. The “triple-negative” phenotype, as defined by immunohistochemistry, encompasses biologically distinct tumors with meaningful differences in immune features, signaling dependencies, genomic alterations, and mesenchymal or stromal programs, which in turn drive heterogeneous responses to uniform therapeutic strategies. In this context, the most informative trials do not rely exclusively on a single conventional biomarker, such as PD-L1 or germline BRCA1/2, but integrate immunohistochemical, transcriptomic and genomic features into treatment-allocation algorithms, functioning as prospective platforms for identifying actionable vulnerabilities across a broader spectrum of patients. Key clinical trials incorporating subtype-guided strategies in metastatic TNBC are summarized in Table 2.

A particularly well-structured example is the program developed at the Fudan University Shanghai Cancer Center, which proposed four biologically and therapeutically relevant subtypes—Luminal Androgen Receptor (LAR), Immunomodulatory (IM), Basal-like Immune-Suppressed (BLIS), and Mesenchymal-like (MES)—and subsequently translated this classification into a prospective therapeutic framework [18,107]. The FUTURE trial, a multicenter, open-label, phase II umbrella study, represents one of the first prospective proof-of-concept demonstrations that a subtype-guided strategy is feasible and capable of generating clinically meaningful efficacy signals in a highly challenging clinical setting. A total of 141 patients with heavily pretreated mTNBC were enrolled, with a median of three prior lines of therapy in the metastatic setting. In the final analysis, confirmed objective responses were observed in 42 patients, corresponding to an overall ORR of 29.8% (95% CI, 22.4–38.1), with a median PFS of 3.4 months (95% CI, 2.7–4.2) and a median OS of 10.7 months (95% CI, 9.1–12.3). These results compare favorably with historical late-line chemotherapy benchmarks, although the noncomparative design precludes definitive conclusions regarding superiority [107].

Beyond its overall results, the true significance of FUTURE lies in its ability to dissect efficacy according to specific biological subpopulations and to show which combinations may be better suited to each molecular context. The study evaluated seven parallel arms and used Bayesian predictive probability rules that allowed closure for futility or efficacy. Four arms met prespecified efficacy boundaries—A, C, E and G—supporting the concept that molecular subtyping can guide therapeutic allocation with differential clinical yield [107].

In the IM subtype, the arm based on PD-1 blockade plus chemotherapy, camrelizumab plus nab-paclitaxel, was the most notable. It included 46 patients and achieved an ORR of 43.5% (95% CI, 28.9–58.9), with a median PFS of 4.6 months (95% CI, 3.4–5.9) and a median OS of 16.1 months (95% CI, 11.7–20.5). Among patients with confirmed responses, the median duration of response was 8.6 months. These findings reinforce a central principle of molecular subclassification: selecting immune-enriched tumors for immunotherapeutic strategies may increase the likelihood of benefit in later treatment lines, a goal that has historically been difficult to achieve in refractory TNBC [107].

In the BLIS subtype without germline BRCA1/2 mutation, an anti-VEGF/VEGFR-based arm enrolled 46 patients and produced 13 confirmed responses, including one complete response and 12 partial responses, yielding an ORR of 28.3% (95% CI, 16.0–43.5), a median PFS of 3.4 months (95% CI, 1.7–5.0), and a median OS of 10.1 months (95% CI, 3.8–16.3). Although this result was less striking than that observed in the IM subtype, it is biologically consistent with the immune-suppressed and angiogenesis-associated features attributed to BLIS tumors and suggests that remodeling the microenvironment through the angiogenic axis may represent a pragmatic therapeutic strategy in this subgroup [18,107].

In a rare but biologically actionable subgroup, patients with LAR tumors harboring ERBB2 mutations received pyrotinib plus capecitabine. Although highly hypothesis-generating because only four patients were enrolled, three confirmed responses were observed, corresponding to an ORR of 75%, with a median PFS of 3.4 months and a median OS of 16.7 months. This result illustrates how subtype-guided genomic profiling can uncover actionable ERBB2 mutations within tumors classified as HER2-negative by conventional criteria, thereby enabling targeted strategies that would not be apparent on the basis of standard immunohistochemistry alone [107].

In the MES subtype with PI3K/AKT mutation, the arm evaluating everolimus plus nab-paclitaxel enrolled nine patients, with three confirmed responses, a median PFS of 3.0 months and a median OS of 4.5 months. Although the signal was modest and the prognosis of advanced MES disease remained poor, this very small exploratory cohort suggests that mTOR inhibition may have context-dependent activity shaped by the broader biological setting rather than by the isolated presence of a molecular alteration [107].

Equally important, FUTURE also documented arms with insufficient activity. In the LAR subtype without ERBB2 mutation, strategies based on androgen-axis inhibition did not yield confirmed responses and were associated with a short median PFS of approximately 1.9 months. This observation suggests that, in heavily pretreated disease, AR expression may sometimes function more as a phenotypic marker than as a sufficient therapeutic dependency, or alternatively that dependency on the androgen axis may be lost over the course of clonal evolution under treatment pressure. This negative result is particularly informative, as it underscores that subclassification not only identifies therapeutic opportunities but also defines biological limits and helps avoid overly simplistic extrapolations [107].

Building on this proof of concept in refractory disease, the program subsequently evolved toward settings with greater potential for clinical impact, particularly the first-line metastatic setting. FUTURE-SUPER is a phase II, open-label, randomized umbrella study in which patients are categorized into cohorts according to subtype and genomic biomarkers and then assigned 1:1 to receive nab-paclitaxel alone or nab-paclitaxel combined with a subtype-guided strategy: pyrotinib in LAR-ERBB2-mutant tumors, everolimus in tumors with PI3K/AKT pathway alterations, camrelizumab plus famitinib in the IM subtype, and bevacizumab in BLIS/MES tumors without PI3K/AKT pathway alterations. In the reported analysis, which included 139 randomized patients with a median follow-up of 22.5 months, the subtype-guided strategy achieved a median PFS of 11.3 months versus 5.8 months in the control arm (HR 0.44; 95% CI, 0.30–0.65; *p* < 0.0001), with a safety profile consistent with combinations incorporating targeted therapy, immunotherapy and antiangiogenic agents. The most common grade 3–4 treatment-related adverse events were neutropenia, anemia and increased alanine aminotransferase, and no treatment-related deaths were reported [13].

These results suggest that molecular subtyping may be clinically useful not only as an exploratory framework, but also as a treatment-optimization strategy in the first-line setting, allowing therapies to be assigned rationally according to the dominant biological features of each tumor rather than applying a single uniform approach across the entire TNBC spectrum. Nevertheless, FUTURE-SUPER should be interpreted in the context of its phase II design and its control arm, which may not fully reflect all contemporary first-line standards for PD-L1-positive mTNBC [13].

Consistent with a cascade development model from umbrella signal detection to confirmatory testing, these findings have supported the development of several phase III subtype-specific trials, including NCT05760378 in IM disease, evaluating famitinib plus camrelizumab plus physician’s choice chemotherapy versus camrelizumab plus chemotherapy; NCT05806060 in BLIS disease, evaluating VEGFR BP102 plus chemotherapy versus chemotherapy; and NCT05954442 in LAR tumors with PI3K/AKT/mTOR pathway alterations, evaluating everolimus plus chemotherapy versus chemotherapy. Taken together, the FUTURE program provides one of the strongest prospective arguments to date for considering molecular subtyping as a composite, actionable biomarker capable of guiding treatment allocation. However, definitive integration into routine clinical practice will require confirmation from ongoing randomized phase III trials [108,109,110].

## 6. Barriers to Clinical Implementation

### 6.1. Technical and Logistical Limitations of Molecular Profiling

The clinical implementation of molecular subclassification in TNBC faces multiple technical and logistical barriers that limit its widespread adoption. Although classification systems based on gene expression profiling have substantially improved our understanding of TNBC heterogeneity, their clinical utility remains constrained by the complexity of the required platforms and by turnaround times.

The development of immunohistochemistry (IHC)-based classifiers using markers such as AR, CD8, FOXC1, and DCLK1 has facilitated the potential practical application of molecular subtyping, showing substantial concordance with mRNA-based classification and independent prognostic value for relapse-free survival [111]. Importantly, surrogate IHC-based approaches may represent the most realistic bridge between molecular subclassification and immediate clinical implementation. Unlike transcriptomic platforms, IHC is inexpensive, widely available in pathology laboratories, compatible with formalin-fixed paraffin-embedded tissue, and already integrated into routine diagnostic workflows. Panels incorporating markers such as AR, CD8, FOXC1, DCLK1, EGFR, basal cytokeratins, Ki-67, PD-L1, and other immune markers can provide clinically meaningful information on androgen dependence, immune infiltration, basal-like biology, mesenchymal differentiation, proliferative activity, and potential therapeutic vulnerabilities [111,112].

From a pragmatic perspective, IHC-based subtyping could be particularly valuable in settings where access to RNA sequencing, broad NGS panels, or multi-omic platforms is limited. It may reduce turnaround time, facilitate decentralized molecular stratification, and allow repeated assessment across disease evolution using metastatic biopsies. These features are especially relevant in metastatic TNBC, where therapeutic decisions often need to be made rapidly and where full transcriptomic profiling may not be available in routine clinical practice. However, IHC still has important limitations in terms of interlaboratory reproducibility, antibody selection, scoring methods, and standardization of cutoff values [112]. Therefore, IHC should not be viewed as a complete substitute for comprehensive molecular profiling, but rather as a cost-effective and clinically accessible intermediate layer capable of translating TNBC biology into routine practice, provided that prospective validation and external quality-control procedures are implemented.

Next-generation sequencing (NGS) platforms, in turn, allow identification of actionable genomic alterations, but their implementation requires specialized infrastructure, trained personnel, and turnaround times that are not always compatible with the urgency of clinical decision-making in metastatic disease [113]. In one real-world study, only 9% of patients with metastatic breast cancer had their treatment strategy modified on the basis of NGS results [113]. In addition, circulating tumor DNA (ctDNA)-based assays, although advantageous for capturing tumor heterogeneity, show variable sensitivity, particularly in samples with low tumor fraction (<1%) [114,115].

Turnaround time is another relevant obstacle. In the FUTURE-SUPER trial, molecular results were delivered to the tumor board within a median of 14 days (range, 11–22 days), a reasonable interval in the first-line setting but potentially problematic in scenarios of rapidly progressive disease or visceral crisis [13,116]. This is further compounded by the need for repeated biopsies to characterize metastatic disease, with the associated logistical burden, financial cost, and clinical risk.

### 6.2. Discordance Between Molecular Subtypes and Approved Biomarkers (PD-L1, BRCA, TILs)

There is significant discordance between TNBC molecular subtypes and the biomarkers currently approved for therapeutic selection, complicating the integration of both systems into routine clinical practice.

PD-L1 expression shows a moderate correlation with stromal TILs (rs = 0.502; *p* < 0.001), but not all tumors with high immune infiltration express PD-L1, and not all PD-L1-positive tumors display a truly inflamed microenvironment [117]. Indeed, TIL-negative/PD-L1-positive and TIL-positive/PD-L1-negative tumors do not represent classical “hot” phenotypes; both are associated with poorer prognosis and lower immunotherapy efficacy compared with TIL-positive/PD-L1-positive tumors [117]. Genomic alterations, including PD-L1 copy number changes and activation of oncogenic pathways, may explain these paradoxical dissociations between TILs, PD-L1, and prognosis [118].

The IM subtype has the highest proportion of patients with PD-L1 positivity and high TIL infiltration, whereas the LAR subtype is characterized by lower immune infiltration, reduced immune checkpoint expression, and low HRD scores [17,48,51]. However, clinical guidelines recommend pembrolizumab plus chemotherapy solely on the basis of PD-L1 CPS ≥10, without routinely integrating molecular subtype information [119].

Germline BRCA1/2 mutations likewise do not directly determine molecular subtype, although they are more common in basal-like tumors. One study showed that BRCA mutations do not directly determine the extent or organization of TILs in TNBC, although the BRCA-mutated group had a significantly higher incidence of tumors with high lymphocytic infiltration than the BRCA wild-type group (*p* = 0.037) [120]. Altogether, this suggests that BRCA biomarkers and molecular subtypes provide complementary, but not interchangeable, information.

In this setting, the integration of multiple biomarkers appears necessary to optimize treatment selection. PD-L1-positive tumors are more frequently clustered within immune-high and Basal-like 1-high subtypes, whereas PD-L1-zero tumors are enriched in LAR-high and mesenchymal-high subtypes [121]. This imperfect correlation between approved biomarkers and molecular subtypes underscores the need for more complex, integrated clinical decision algorithms.

### 6.3. Temporal Stability and Subtype Evolution Across Treatment Lines

The temporal stability of TNBC molecular subtypes and their behavior under therapeutic pressure represent a critical concern for the clinical application of subtype-guided strategies.

A study from the Pan-Pacific TNBC Consortium demonstrated that 56% of TNBC tumors changed molecular subtype after neoadjuvant chemotherapy among patients who did not achieve pathological complete response. The most frequent shift was from BL1 to a mesenchymal subtype (38%), whereas no tumors changed from M to BL1. The immunomodulatory signature was positive in 22% of patients before treatment and in only 12.5% after chemotherapy [122].

This subtype switching was closely associated with epithelial–mesenchymal transition. The EMT score increased after chemotherapy in 78% of patients with subtype change, compared with only 39% of those without subtype change (*p* = 0.002) [122]. These data suggest that chemotherapy may select resistant cellular populations and/or promote transcriptional reprogramming toward mesenchymal-like phenotypes, potentially contributing to reduced treatment sensitivity.

Therapeutic pressure may also reshape the mutational landscape. Preliminary, hypothesis-generating data from paired pre- and post-treatment TNBC samples showed that the average tumor mutational burden was 18.15 in primary tumors with pathological complete response, 26.52 in primary tumors without response, and 19.69 in residual tumors. Mutational spectra also appeared to change, with predominance of C>A transversions in tumors achieving complete response and a progressive increase in C>T transitions in non-responding tumors and residual disease [31].

Discordance between primary tumors and metastatic lesions further complicates static biomarker interpretation. Although relatively high concordance between primary and metastatic samples has been reported for selected driver alterations such as TP53 mutations and ERBB2 amplification, clinically relevant discordances can occur in other genes [123]. In broader breast cancer cohorts, paired primary-metastatic analyses have also shown that intrinsic subtype conversion can occur. In a study of 123 paired primary and metastatic tissue samples, subtype conversion rates were 0% in basal-like tumors, 23.1% in HER2-enriched tumors, 30.0% in luminal B tumors, and 55.3% in luminal A tumors. In addition, metastatic tumors were enriched in genes related to proliferation and migration, with reduced expression of luminal genes [124].

These observations have direct clinical implications: subtyping performed on the primary tumor may fail to adequately reflect the biological features of metastatic or residual disease, particularly after exposure to systemic therapy. This may compromise the efficacy of targeted therapies selected on the basis of outdated biological information. Although clinical guidelines recommend biopsy of recurrent or metastatic disease when feasible to confirm diagnosis and reassess clinically actionable biomarkers, systematic molecular re-subtyping at each treatment line still faces major logistical, economic and technical barriers [119,123].

Taken together, the clinical application of molecular subtype-guided therapy in metastatic TNBC is hindered by three major limitations: the technical complexity of molecular profiling, its imperfect concordance with currently approved biomarkers, and the temporal instability of subtypes under therapeutic pressure. These limitations suggest that molecular subclassification should not be interpreted as a static, one-time diagnostic label, but rather as a dynamic biological framework that may evolve throughout the disease course. Overcoming these barriers will require more accessible diagnostic platforms, standardized surrogate approaches such as validated IHC-based panels, integrated biomarker algorithms, and longitudinal monitoring strategies incorporating metastatic re-biopsy and ctDNA analysis when clinically feasible [111,112,113,114,115,116,119,122,123,124].

## 7. Conclusions

Metastatic triple-negative breast cancer remains one of the most complex scenarios in contemporary medical oncology, not only because of its aggressive clinical behavior and poor prognosis, but also because of the longstanding difficulty in accurately identifying which patients are most likely to benefit from each therapeutic strategy. Although the classical definition based on the absence of ER, PR, and HER2 expression remains clinically useful, it is biologically insufficient. This receptor-negative category encompasses a heterogeneous group of tumors with distinct genomic, transcriptomic, epigenetic, immunologic, stromal, and biomechanical features.

As discussed throughout this review, metastatic TNBC should not be regarded as a single disease entity, but rather as a spectrum of biologically distinct states with different molecular dependencies and therapeutic vulnerabilities. The classical Lehmann subtypes—BL1, BL2, M, MSL, IM, and LAR—remain highly informative for understanding tumor biology, whereas clinically oriented systems such as the Fudan classification provide a more directly translational framework for subtype-guided therapeutic development. Importantly, these systems are complementary rather than interchangeable, and approximate cross-mapping between them should be interpreted with caution.

The available evidence supports several clinically relevant principles. Basal-like tumors, particularly BL1, are generally more sensitive to chemotherapy and DNA damage-based strategies, especially when homologous recombination deficiency or BRCA-associated biology is present. Immune-enriched and immunomodulatory tumors represent the most rational candidates for immunotherapy-based approaches. LAR tumors require refined biomarker selection based on androgen receptor pathway activity rather than androgen receptor immunohistochemistry alone, and may benefit from rational combinations targeting PI3K/AKT/mTOR or cell-cycle pathways. Mesenchymal and mesenchymal stem-like tumors remain among the most therapeutically challenging phenotypes, owing to EMT, stromal activation, extracellular matrix remodeling, mechanotransduction, immune exclusion, and adaptive resistance.

The therapeutic landscape of mTNBC is also evolving beyond classical subtype-specific approaches. Antibody–drug conjugates, including TROP-2-directed and HER2-low-directed strategies, have introduced a cross-subtype therapeutic paradigm in which target antigen expression, payload sensitivity, bystander effect, and tumor microenvironmental accessibility may be as relevant as transcriptomic subtype. These agents should therefore be considered complementary to molecular subclassification rather than independent from it.

At the same time, emerging technologies such as single-cell RNA sequencing, spatial transcriptomics, ctDNA analysis, and lncRNA profiling are reshaping our understanding of TNBC heterogeneity. These approaches highlight that TNBC biology is dynamic, spatially organized, and shaped by treatment-driven selective pressure. A single molecular assessment performed at diagnosis may therefore fail to capture the evolving biology of metastatic or residual disease. Future precision oncology strategies will likely require longitudinal characterization, integration of metastatic biopsies when feasible, and dynamic monitoring of clonal evolution and resistance.

Despite this progress, routine implementation remains challenging. Full transcriptomic or multi-omic profiling is not universally available, can be costly, and may not always provide results within clinically actionable timeframes. In this context, validated surrogate immunohistochemistry-based classifiers may offer a practical and cost-effective bridge between molecular biology and immediate clinical practice, particularly if standardized scoring systems and prospective validation are achieved.

Overall, molecular subclassification of metastatic TNBC represents a major step toward biologically informed precision oncology. However, its clinical value will depend on the integration of multiple layers of information—molecular subtype, validated biomarkers, immune contexture, stromal architecture, ADC target expression, prior treatment exposure, and patient-specific clinical factors—into pragmatic and reproducible decision-making models. The future management of mTNBC will likely move from a uniform chemotherapy-based approach toward a dynamic, subtype-informed, biomarker-integrated strategy capable of improving outcomes in a patient population that has historically derived limited benefit from therapeutic innovation.

## Figures and Tables

**Table 1:** Summary of the main molecular subtypes of metastatic triple-negative breast cancer, including their biological features, dominant signaling programs, representative biomarkers, clinical behavior, potential therapeutic implications, and approximate correspondence with clinically oriented classification systems.

Subtype	Key bA1.	Dominant Pathways	Biomarker Alterations	Clinical Behavior	Potential Therapeutic Implications	Approximate Correspondence with Fudan/Burstein Systems
Basal-like 1 (BL1)	Highly proliferative and genomically unstable tumors with prominent DNA damage response activity.	Cell cycle regulation, DNA damage response, homologous recombination deficiency, MYC and WNT signaling.	TP53 mutations, BRCA1/2 alterations, PTEN loss, high Ki-67, HRD phenotype.	Aggressive phenotype, but relatively high sensitivity to chemotherapy, particularly DNA-damaging agents.	Platinum-based chemotherapy; PARP inhibitors in BRCA-mutated or HRD tumors; prioritization of DNA-damaging strategies.	Approximate overlap with Fudan BLIS or basal/proliferative tumors; may correspond to Burstein BLIA or BLIS depending on immune activation.
Basal-like 2 (BL2)	Growth factor-driven subtype with lower chemosensitivity than BL1 and greater stromal influence.	RTK signaling, EGFR, MET, FGFR, TGFβ, CXCL8, and PI3K/AKT-related signaling.	AKT1 overexpression, copy number gains, RTK activation signatures.	Poorer prognosis and lower response to standard chemotherapy compared with BL1.	Investigational use of RTK inhibitors, FGFR-directed strategies, and PI3K/AKT-targeted approaches.	Partial overlap with Fudan BLIS or growth factor-enriched basal tumors; no exact equivalent in the Fudan system.
Mesenchymal (M)	EMT-enriched, invasive, stromal-rich tumors with marked immune evasion.	EMT, extracellular matrix remodeling, RTK-RAS signaling, PI3K/mTOR, PRC2-mediated repression, mechanotransduction.	EMT markers, angiogenesis signatures, copy number losses in immune-related regions, low PD-L1 expression.	Chemoresistant, highly invasive, and generally associated with poor prognosis.	PI3K/mTOR inhibitors; antiangiogenic agents; stromal-targeting combinations; investigational PRC2 or mechanotransduction-directed strategies.	Closest correspondence with Fudan MES and Burstein MES subtypes.
Mesenchymal Stem-like (MSL)	Stem-like and stromal-influenced phenotype with mesenchymal, angiogenic, and microenvironment-derived features.	EGFR signaling, RTK-RAS pathway, Wnt/β-catenin, Wnt/PCP, angiogenesis programs, stromal interaction.	CD44, ALDH1A1, EGFR-related signatures, angiogenesis-associated profiles.	Lower pathological complete response rates, intermediate chemosensitivity, and poorer long-term outcomes.	PI3K/mTOR inhibitors; SRC/ABL-directed approaches such as dasatinib; antiangiogenic strategies; combinations aimed at reversing EMT or targeting stromal dependencies.	Partial correspondence with Fudan MES/stromal-enriched tumors; often interpreted as a microenvironment-influenced rather than purely tumor-intrinsic subtype.
Immunomodulatory (IM)	Immune-enriched subtype with adaptive immune activation and dense lymphocytic infiltration.	PD-1/PD-L1 axis, CTLA-4, interferon-α/γ signaling, antigen presentation, Notch pathway.	PD-L1 expression, CD8+ infiltration, high TILs, immune checkpoint gene expression.	Better prognosis relative to other subtypes and enhanced sensitivity to immunotherapy.	Immune checkpoint inhibitors combined with chemotherapy; potentially antiangiogenic-immunotherapy combinations in selected immune-rich tumors.	Closest correspondence with Fudan IM and Burstein immune-activated/BLIA-like tumors.
Luminal Androgen Receptor (LAR)	Androgen-driven luminal-like subtype with low proliferation and distinct endocrine-like biology.	AR signaling, FOXA1, ERBB2-related signaling, PI3K/AKT, JAK-STAT.	AR expression, PIK3CA mutations, AKT1 mutations, CDH1 mutations, low HRD score.	Low sensitivity to chemotherapy, tendency toward bone and nodal metastases, and variable overall prognosis.	Antiandrogen strategies in tumors with high AR pathway activity; combination approaches with PI3K/AKT/mTOR or cell-cycle inhibitors.	Shared across Lehmann, Fudan, and Burstein classifications as the androgen receptor-driven luminal-like subtype.

Abbreviations: AR, androgen receptor; BLIA, basal-like immune-activated; BLIS, basal-like immune-suppressed; EMT, epithelial–mesenchymal transition; Fudan MES, mesenchymal-like subtype in the Fudan classification; HRD, homologous recombination deficiency; IM, immunomodulatory; LAR, luminal androgen receptor; MSL, mesenchymal stem-like; PARP, poly(ADP-ribose) polymerase; RTK, receptor tyrosine kinase; TILs, tumor-infiltrating lymphocytes. The correspondence between classification systems should be interpreted as approximate, because Lehmann, Fudan, and Burstein classifications are based on related but non-identical transcriptomic frameworks.

**Table 2:** Key clinical trials evaluating subtype-guided therapeutic strategies in metastatic triple-negative breast cancer.

Subtype/Population	Trial	Therapeutic Strategy	Key Results	Clinical Interpretation
IM/immune-enriched mTNBC	FUTURE	Camrelizumab plus nab-paclitaxel	ORR 43.5%; median PFS 4.6 months; median OS 16.1 months.	Supports sensitivity of immune-enriched tumors to immunotherapy-based combinations, even in heavily pretreated disease.
IM/first-line mTNBC	FUTURE-SUPER	Camrelizumab, famitinib, and nab-paclitaxel	Median PFS 15.1 months; HR 0.46 versus chemotherapy alone.	Strongest signal for subtype-guided immunotherapy and antiangiogenic combination strategies in immune-enriched disease.
BLIS/BRCA wild-type mTNBC	FUTURE	Anti-VEGF/VEGFR-based therapy	ORR 28.3%; median PFS 3.4 months.	Suggests potential clinical activity of angiogenesis-targeted strategies in immune-suppressed basal-like tumors.
BLIS/MES PI3K/AKT wild-type/first-line mTNBC	FUTURE-SUPER	Bevacizumab plus nab-paclitaxel	Median PFS 9.1 months versus 3.9 months; HR 0.35.	Supports antiangiogenic therapy as a rational approach in selected BLIS/MES tumors without PI3K/AKT pathway alterations.
LAR with ERBB2 mutation	FUTURE	Pyrotinib plus capecitabine	ORR 75%; small cohort.	Illustrates the ability of subtype-based profiling to identify actionable HER2 mutations in tumors classified as HER2-negative by standard criteria.
LAR with PI3K/AKT alteration/first-line mTNBC	FUTURE-SUPER	Everolimus plus nab-paclitaxel	Median PFS 13.9 months versus 6.1 months; HR 0.48.	Suggests potential benefit of PI3K/AKT/mTOR pathway targeting in molecularly selected LAR tumors, although subgroup size was limited.
MES with PI3K/AKT alteration	FUTURE	Everolimus plus nab-paclitaxel	Median PFS 3.0 months; limited OS benefit.	Indicates context-dependent and modest activity of mTOR inhibition in heavily pretreated mesenchymal-like disease.
All randomized subtype-defined cohorts/first-line mTNBC	FUTURE-SUPER	Subtype-guided therapy plus nab-paclitaxel versus nab-paclitaxel alone	Median PFS 11.3 versus 5.8 months; HR 0.44; *p* < 0.0001.	Provides proof-of-concept that subtype-guided treatment allocation can improve first-line outcomes in mTNBC.

Abbreviations: BLIS, basal-like immune-suppressed; BRCA, breast cancer susceptibility gene; ERBB2, erb-b2 receptor tyrosine kinase 2; HR, hazard ratio; IM, immunomodulatory; LAR, luminal androgen receptor; MES, mesenchymal-like; mTNBC, metastatic triple-negative breast cancer; ORR, objective response rate; OS, overall survival; PFS, progression-free survival; VEGF, vascular endothelial growth factor; VEGFR, vascular endothelial growth factor receptor.

## Data Availability

No new data were created or analyzed in this study. Data sharing is not applicable to this article.
